# Supplementary material for: Latent Human Cytomegalovirus Infection Activates the STING Pathway but p-IRF3 Translocation Is Limited
Source: Viruses. 2025 Aug 12;17(8):1109. doi: 10.3390/v17081109 (PMC12390657; doi:10.3390/v17081109)
Supplement: Supplementary file 1 [file viruses-3672478-supplementary.pdf › viruses-3672478-supplementary.pdf]

Suppl. Fig. 1

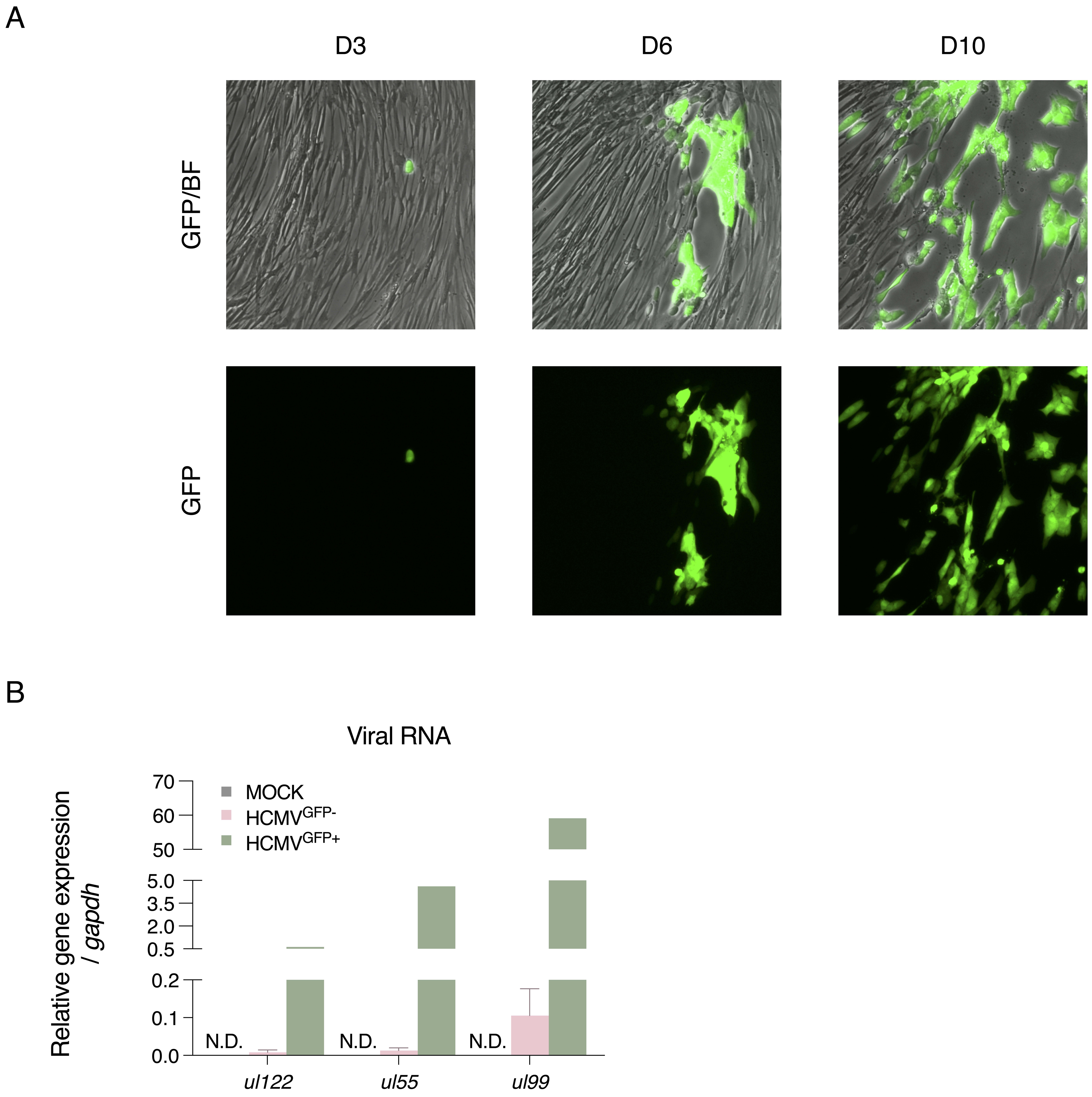

Suppl. Fig. 1 HCMV RV-TB40-BAC$_{KL7}$-SE-EGFP infected cell morphology and gene expression in productive and latent infection. (A) RV-TB40-BAC$_{KL7}$-SE-EGFP forms GFP^{+} plaques on Hs27 fibroblasts over time. Photo taken with 40X magnification. (B) RV-TB40-BAC$_{KL7}$-SE-EGFP expresses different gene profiles in productive (HCMV^{GFP+}) and latent (HCMV^{GFP-}) cells. qPCR analysis of relative viral RNAs, *ul122, ul55* and *ul99*, expression level on day 7 post-infection (n=3 for HCMV^{GFP-}; n=1 for HCMV^{GFP+}). *gapdh* served as the house-keeping gene control. Graphs represent the mean ± SEM.

Suppl. Fig. 2

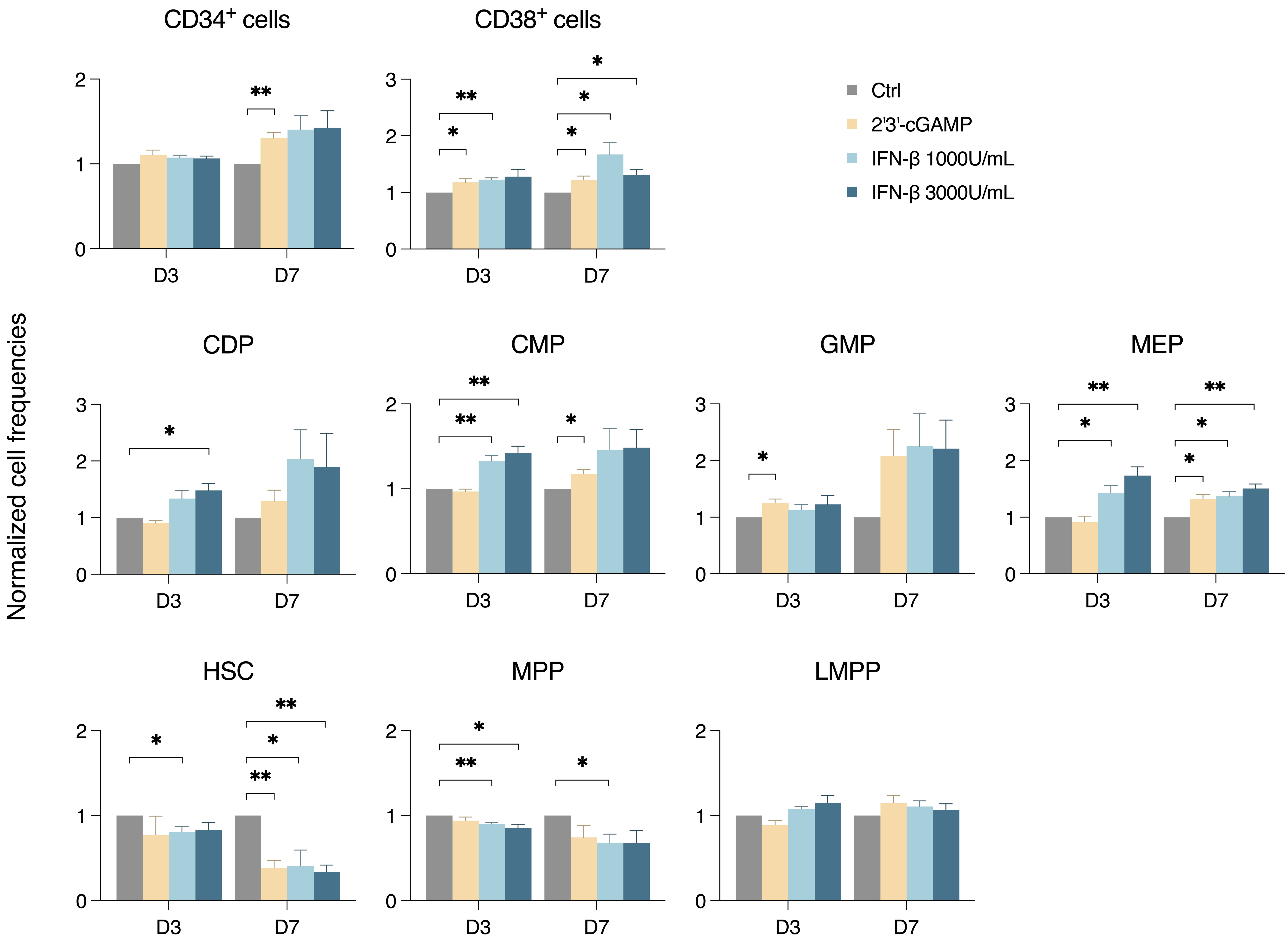

Suppl. Fig. 2 IFN-β or 2’3’-cGAMP treatment of CD34$^+$ cells induced differentiation into CD38$^+$ myeloid progenitor subsets over time. CD34$^+$ cells were treated with 1000 U/mL, 3000 U/mL IFN-β or 100 µg/mL 2’3’-cGAMP for 3 and 7 days. Differentiation of the subsets was examined by multiparameter flow cytometry. Analysis of CD34$^+$ progenitor subsets frequencies normalized to untreated control (n=3). Graphs show the mean ± SEM from three independent experiments. *P<0.05, **P<0.01.

Suppl. Fig. 3

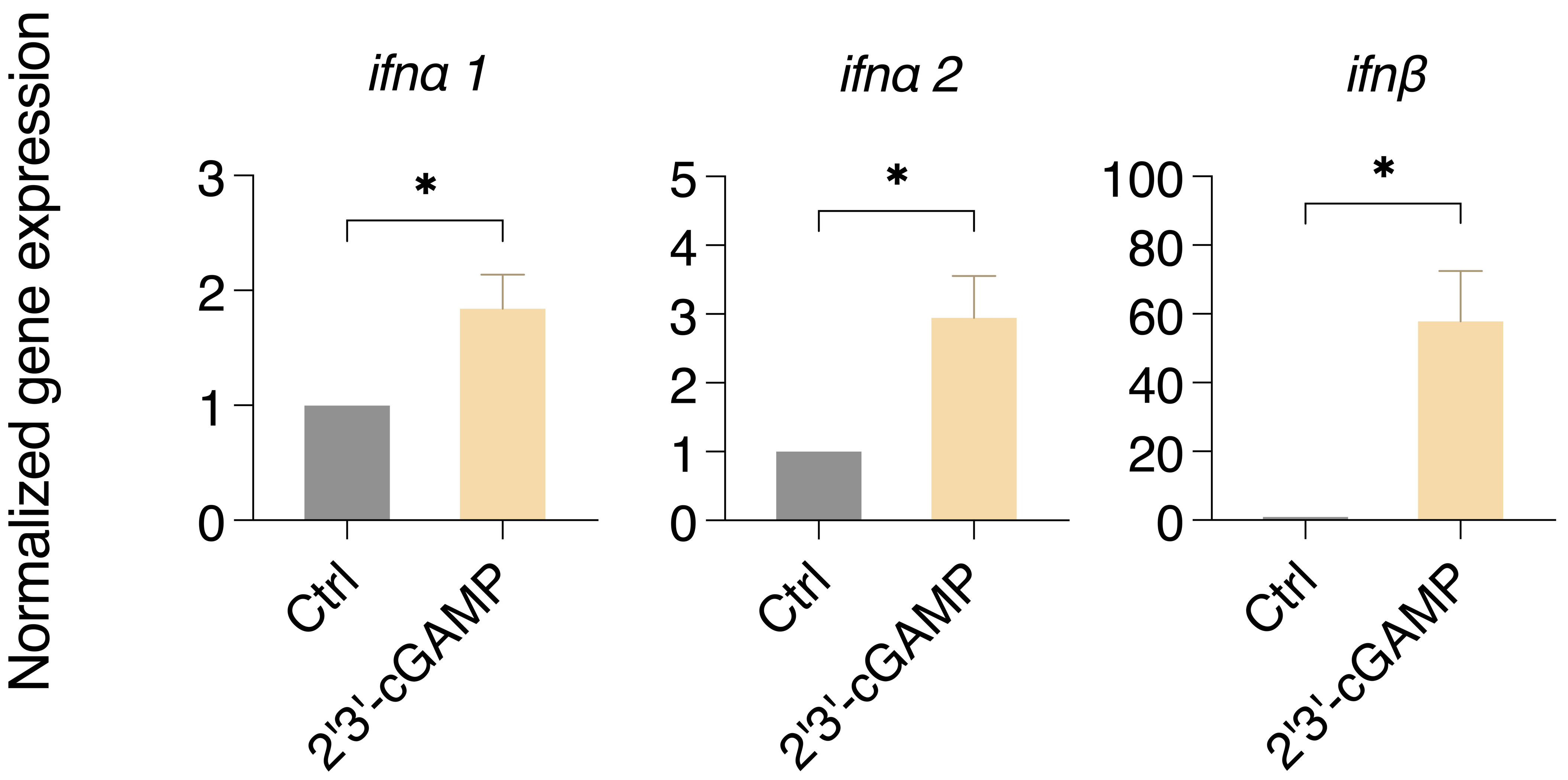

Suppl. Fig. 3 Gene expression level of type I IFNs by CD34^{+} cells after 2'3'-cGAMP stimulation. CD34^{+} cells were treated with 100 µg/mL 2'3'-cGAMP to activate the STING pathway. Cells were collected on 4 hrs for qPCR analysis of *ifna1, ifna2* and *ifnb1* normalized to control (n=3). *gapdh* served as the house-keeping control. Graphs show the mean ± SEM from data of three independent experiments.

Suppl. Fig. 4

A

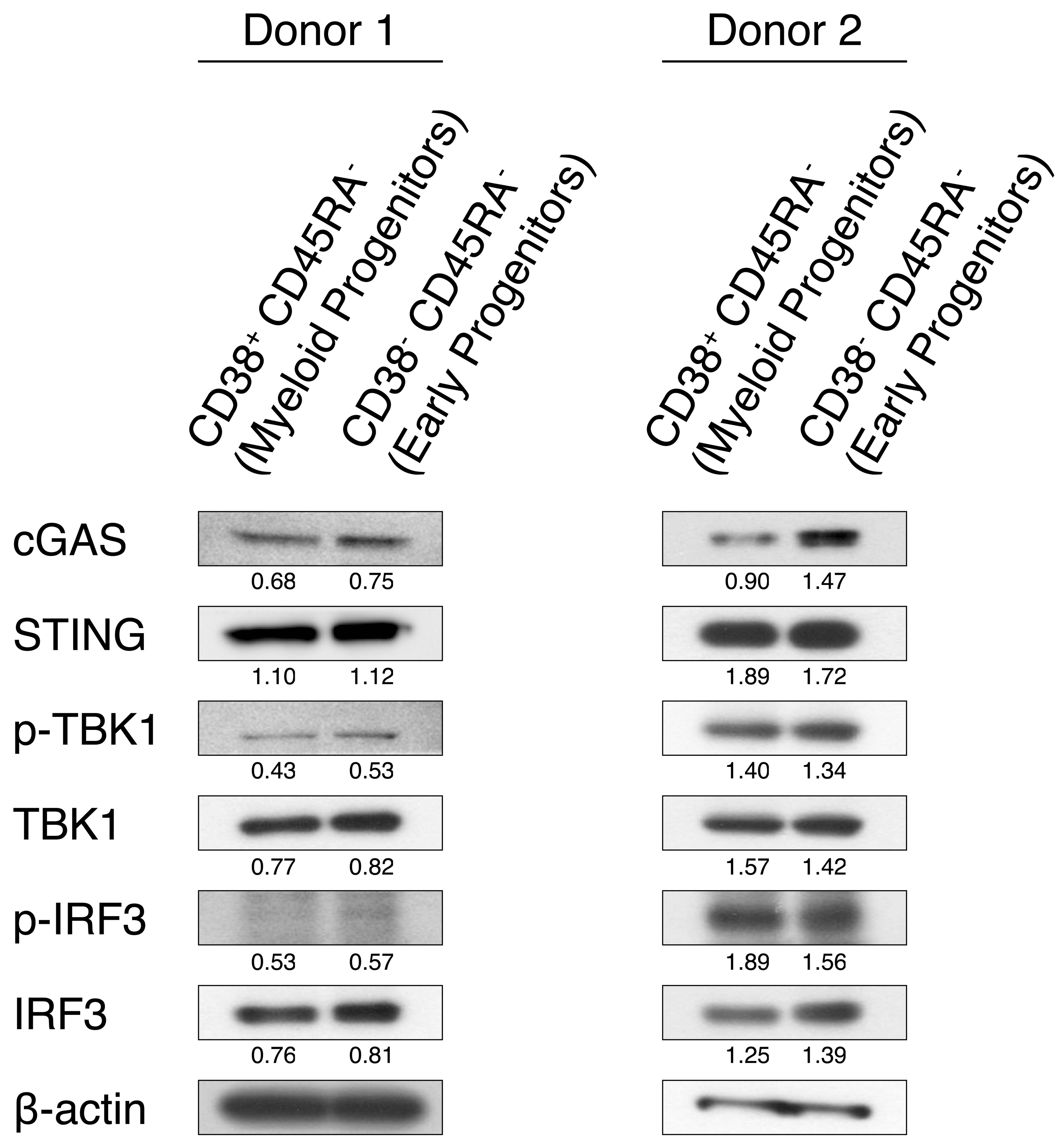

B

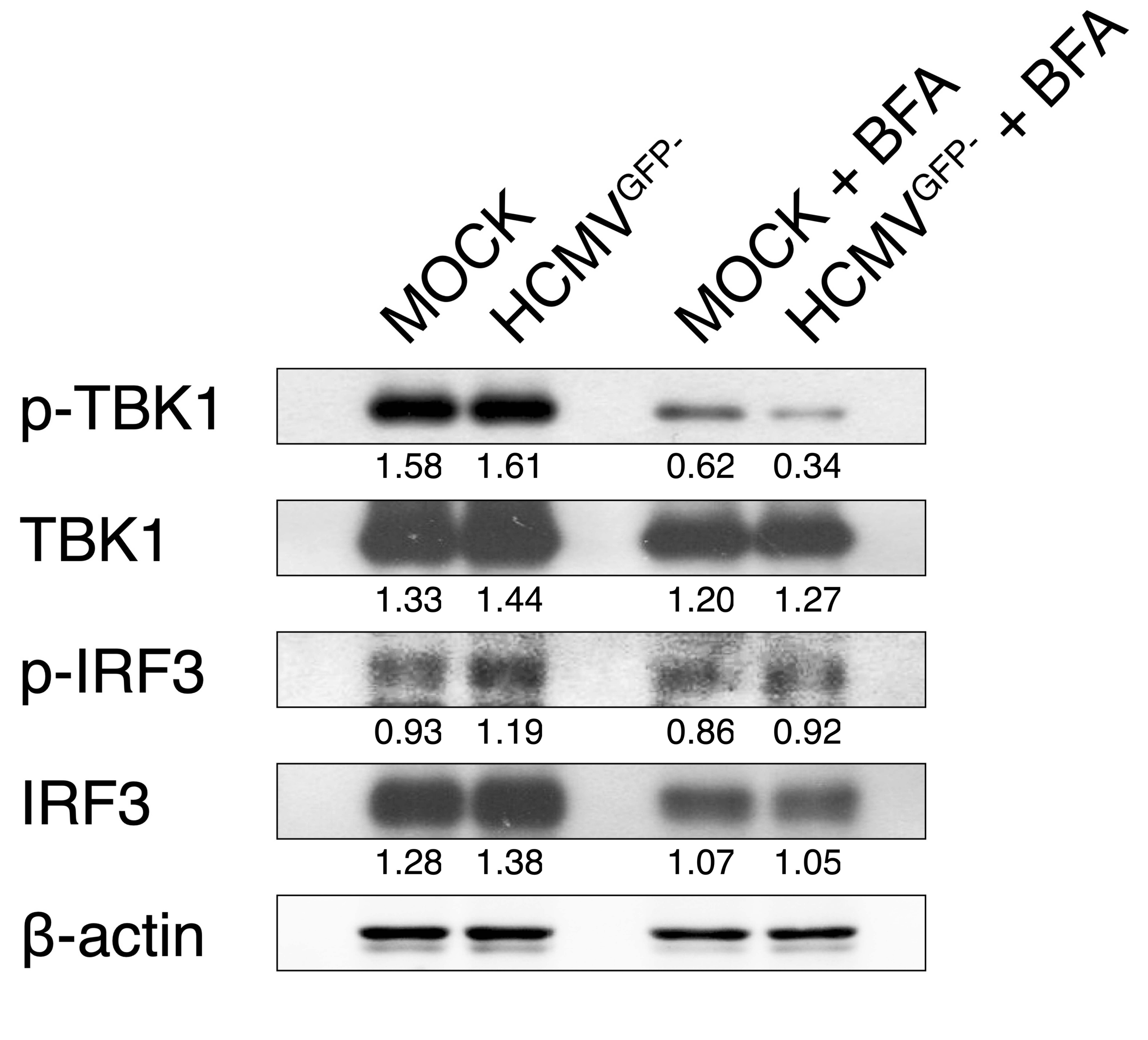

Suppl. Fig. 4 Western blot analysis of STING pathway proteins in CD34$^+$ early and myeloid progenitors, and BFA treatment on the MOCK and HCMV^{GFP-} cells. (A) CD34$^+$ cells were immunostained and FACSorted with the myeloid (CD38$^+$CD45RA$^-$) and early (CD38$^-$CD45RA$^-$) markers for Western blotting analysis of cGAS, STING, p-TBK1, TBK1, p-IRF3 and IRF3. β-actin served as the endogenous control. Cells from two independent donors were tested. (B) The mock and HCMV^{GFP-} cells were treated with BFA for 1 day. Representative western blotting analysis of p-TBK1, TBK1, p-IRF3 and IRF3 was shown for mock and HCMV^{GFP-} cells at day 7 P.I. Numbers under the blot indicate the band intensities for the different proteins normalised to β-actin.

Suppl. Fig. 5

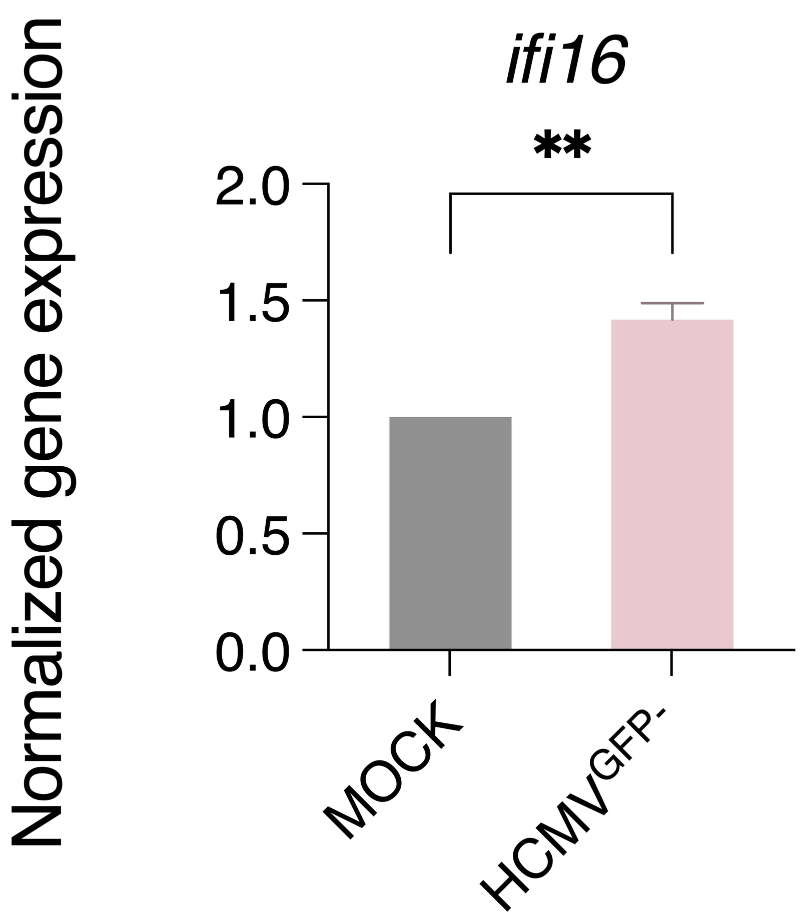

Suppl. Fig. 5 Gene expression of DNA sensor *IFI16* is induced in the latently infected HCMV^{GFP-} cells. Total RNA were extracted from mock and FACSorted HCMV^{GFP-} cells on day 7 post-infection and subjected to RT-qPCR analysis of the expression of *ifi16* normalized to mock (n=3). *gapdh* served as the house-keeping gene control. Data shown represents mean ± SEM from three independent experiments.

Suppl. Table. 1

Suppl. Table 1 Frequencies of $HCMV^{GFP+}$ cells found among cell subsets in HCMV infected cells. (day 1 P.I., n =4)

		SUBSET						
HCMV		**CDP**	**CMP**	**GMP**	**MEP**	**HSC**	**MPP**	**LMPP**
GFP+	Mean	6.66	1.28	1.57	1.76	0.33	0.14	0.05
	SEM	1.36	0.18	0.26	0.51	0.15	0.03	0.01
GFP-	Mean	91.92	98.50	97.70	77.32	99.68	99.84	99.94
	SEM	1.76	0.22	0.40	8.66	0.14	0.03	0.01
